# Prevalence of anemia before and after initiation of antiretroviral therapy among HIV infected patients at Black Lion Specialized Hospital, Addis Ababa, Ethiopia: a cross sectional study

**DOI:** 10.1186/s12878-018-0099-y

**Published:** 2018-03-15

**Authors:** Gashaw Garedew Woldeamanuel, Diresibachew Haile Wondimu

**Affiliations:** 1 Department of Medicine, College of Medicine and Health Sciences, Wolkite University, P.O. Box 07, Wolkite, Ethiopia; 2 Department of Medical Physiology, School of Medicine, College of Health Sciences, Addis Ababa University, Addis Ababa, Ethiopia

**Keywords:** HIV, ART, Anemia, Ethiopia

## Abstract

**Background:**

Anemia is the most common hematological abnormality in Human immunodeficiency virus (HIV) positive patients and a significant predictor of its progression to AIDS or death. This study was aimed to assess the prevalence of anemia before and after initiation of antiretroviral therapy (ART) among HIV positive patients attending Black Lion Specialized Hospital, Addis Ababa, Ethiopia.

**Methods:**

A cross sectional study was conducted from January to April, 2017 in Black Lion Specialized Hospital, Addis Ababa, Ethiopia. A total of 255 patients on ART were selected using simple random sampling techniques. Socio-demographic and clinical characteristics of the study subjects were collected using structured questionnaire. Measurements of complete blood cell counts and CD4 + T cell counts were made using Sysmex XT 2000i hematology analyzer and BD FACS Count CD4 analyzer, respectively. Statistical analysis of the data (Chi-square, paired T-test, logistic regression) was done using SPSS version 20. A *p*-value < 0.05 was considered as significant.

**Results:**

Prevalence of anemia before and after ART initiation was 41.9 and 11.4% respectively. There are a significance differences in CD4 + T cell count, RBC count, hemoglobin values and RBC indices in HIV patients before and after ART initiation (*p*-value < 0.05). WHO clinical stages and CD4+ T cell counts were found to be associated with the prevalence of anemia before ART initiation. Among the total number of anemic cases, normocytic normochromic anemia was present in 71% of the cases before ART and in 58.6% of the cases after ART. The prevalence of macrocytic normochromic anemia before and after ART initiation was 4.7 and 27.6% respectively.

**Conclusions:**

It is evident from this study that there is a remarkable reduction in the prevalence of anemia after ART initiation. However, a significant proportion of HIV patients remained anemic after 6 months of ART initiation suggesting the need for routine screening and proper treatment of anemia to mitigate its adverse effects.

## Background

Hematologic abnormalities are among the most common manifestations of advanced human immunodeficiency virus (HIV) infection and acquired immunodeficiency syndrome (AIDS) [1]. Low blood cell counts, are the most common of these disorders [2]. The frequency and severity of these hematological manifestations increased with the decline in CD4 counts [3] with anemia being the most common hematologic abnormality in HIV patients and is associated with disease progression and decreased survival [4]. However, the prevalence of anemia in HIV patients varies considerably, ranging from 1.3 to 95%. Several factors including stage of HIV, age, sex and the definition of anemia used are said to account for the variations in HIV prevalence [5].

Anemia is multifactorial. HIV infection itself causes anemia, probably as a consequence of HIV infection of stromal cells. Other common causes of anemia in AIDS are anemia of chronic disease, bone marrow suppression by ART, and hemolytic anemia induced by oxidant drugs [6, 7]. Cytokines such as interleukin 1, tumor necrosis factor and the interferon play a role in impairing erythropoietin response by reducing concentration of marrow progenitors and erythroid colonies. As ART generally diminishes these cytokines, anemia is less common than in the pre ART era. But commonly used myelosuppressive drugs in the HIV setting may contribute to anemia and even in the era of ART, anemia continues to contribute to morbidity and diminished quality of life [8].

Overall, the treatment of HIV infection with ART reduces the incidence of anemia and increases hemoglobin levels over time [9]. However, zidovudine (AZT) has also been clearly demonstrated to cause anemia [10]. Marrow erythroid hypoplasia, aplasia, and megaloblastic maturation have developed as a result of AZT therapy [8]. The effect of AZT is modest when taken as ART than administered as a single dose [11].

Different studies were conducted to assess the prevalence of anemia in HIV infected individuals. But there are only few published reports in Ethiopia on the assessment of anemia among HIV positive patients. Therefore, this study gave information about the prevalence of anemia before and after initiation of antiretroviral therapy among HIV patients who attended at ART clinic of Black Lion Specialized Hospital, Addis Ababa, Ethiopia.

## Methods

### Study setting and study population

Cross sectional study design was conducted at ART clinic of Black Lion Specialized Hospital, Addis Ababa, Ethiopia. This hospital is selected based on the availability of patients from all parts of the country as it is referral and specialized teaching hospital in Ethiopia. This study was conducted from January to April, 2017. During the study period, 2675 HIV infected adults were on ART. Among those on ART, a total of 255 HIV infected patients taking ART for at least 6 months were selected randomly and included in this study. Sample size was estimated using a single population proportion formula, taking *p* = 20.9% (expected prevalence rate of anemia) [11], 5% level of precision (d) with 95% confidence interval. The inclusion criteria for this study includes; HIV positive adults greater than 18 years old at the time of ART initiation, those who were on ART for at least 6 months, patients having complete hematological values at the baseline and those who were volunteered to participate in the study. Patients transferred from other health institutions, those who were on medication, pregnant, diagnosed as having hematological diseases and other medical conditions by medical experts were excluded from the study.

### Data collection

The data was collected using a structured questionnaire by five nurses working in the ART clinic. The collected information includes socio-demographics, clinical characteristics, and immunohematological profile of patients at baseline and after 6 months of ART initiation. Data on socio-demographic, clinical characteristics and baseline information of the study participants were collected by interview and review of medical records. Anthropometric measurements were carried out according to the WHO recommendations. Then, the study participants were sent to the laboratory for determination of blood cell counts. Hemoglobin concentration, RBC count and RBC indices were determined using Sysmex XT 2000i hematology analyzer whereas CD4+ T cells were assayed using the BD FACS Count system. The instruments and the procedures used for analysis of blood cell counts were the same during the two time points.

To ensure good quality data, training of data collectors, pre-testing of data collection instrument and continuous supervision of the data collection process were carried out.

### Definition of outcome variable and statistical analysis

The data were cleaned, edited, checked for completeness and entered into SPSS version 20 for analysis. Anemia was defined as Hgb concentration less than 13 g/dl for adult males and less than 12 g/dl for adult females. It was further classified into mild (11–11.9 g/dl for women and 11–12.9 g/dl for men), moderate (8–10.9 g/dl) and severe (< 8 g/dl) for both sexes [12]. Microcytosis was defined as MCV < 80 fl, macrocytosis as MCV > 100 fl and hypochromia was defined as MCHC value < 31 g/dl [13].

Descriptive statistics was used to get a clear picture of dependent and independent variables. After checking the normality of the data, paired T-test was used and also chi square was computed to determine association between dependent and independent variables. Multivariable logistic regression analysis was performed to obtain the adjusted effect of different risk factors on the odds of being anemic at baseline and after 6 months of ART initiation. A *p*-value < 0.05 was considered as statistically significant.

## Results

### General characteristics of study participants

A total of 255 HIV positive patients, of which 148 (58%) women and 107 (42%) men were involved in this study. The overall mean age was 40.6 ± 9.4 years, within the range of 20–70 years old. The majority of patients were within 40–49 years of age. About 95 (37.3%) participants were under WHO clinical stage III and the most widely used ART regimen (62.7%) in this study was 1e (TDF-3TC-EFV). At the time of study, 61.6% of them were taking cotrimoxazole prophylaxis therapy (Table 1).

**Table 1:** Sociodemographic and clinical characteristics of HIV positive patients taking ART at Black Lion Specialized Hospital, Addis Ababa, Ethiopia, 2017

Variables	Frequency (*n* = 255)	Percentage (%)
Age (in years)		
20–29	33	12.9
30–39	86	33.7
40–49	96	37.6
50–59	30	11.8
60–69	9	3.5
70–79	1	4
Sex		
Male	107	42
Female	148	58
Marital Status		
Single	67	26.3
Divorced	43	16.9
Married	106	41.5
Widowed	39	15.3
Educational status		
illiterate	27	10.6
Primary school	101	39.6
High school	91	35.7
Certificate and above	36	14.1
Employment status		
Employed in public organization	26	10.2
Employed in private organization	23	9
Self employed	57	22.4
Unemployed	149	58.4
WHO clinical stages at baseline		
Stage I	41	16.1
Stage II	56	22
Stage III	95	37.3
Stage IV	63	24.6
Types of ART regimens		
1c	40	15.7
1d	30	11.8
1e	160	62.7
1f	25	9.8
Cotrimoxazole prophylaxis		
Yes	157	61.6
No	98	38.4

1c = AZT-3TC-NVP, 1d = AZT-3TC-EFV, 1e = TDF-3TC-EFV, 1f = TDF-3TC-NV

### Red blood cell parameters and CD4^+^ T cell counts of HIV positive patients before and after initiation of ART

There were statistically significant differences in the mean values of RBC count (4.41 ± 0.71 × 10^6^/μl vs. 4.28 ± 0.59 × 10^6^/μl), hemoglobin (12.8 ± 1.99 g/dl vs. 14.34 ± 1.89 g/dl), MCV (86.34 ± 6.42 fl vs. 96.33 ± 8.80 fl), MCH (29.1 ± 2.69 pg vs. 32.78 ± 4.09 pg), MCHC (33.52 ± 1.75 g/dl vs 34.18 ± 1.86 g/dl), RDW (14.91 ± 2.66% vs. 13.66 ± 1.58%) and CD4+ T cell counts (162.5 ± 108.6 cells/μl vs. 347 ± 183.17cells/μl) before and after ART initiation respectively. Patients after ART initiation have high hemoglobin level, MCV, MCH, MCHC and CD4+ T cell counts when compared to ART naïve patients (*p* <  0.001) (Table 2).

**Table 2:** Red blood cell parameters and CD4^+^ T cell counts of HIV positive adult patients at baseline and after 6 months of ART at Black Lion Specialized Hospital, Addis Ababa, Ethiopia, 2017

Parameters	Before initiation of ART (*n* = 255) Mean ± SD	After 6 months of ART initiation (*n* = 255) Mean ± SD	*P*-value
RBC (×10^6^/μl)	4.41 ± 0.71	4.28 ± 0.59	0.009
Hgb (g/dl)	12.8 ± 1.99	14.34 ± 1.89	< 0.001
MCV (fl)	86.34 ± 6.42	96.33 ± 8.80	< 0.001
MCH (pg)	29.1 ± 2.69	32.78 ± 4.09	< 0.001
MCHC (g/dl)	33.52 ± 1.75	34.18 ± 1.86	< 0.001
RDW (%)	14.91 ± 2.66	13.66 ± 1.58	< 0.001
CD4 (Cells/μl)	162.5 ± 108.6	347 ± 183.17	< 0.001

### Prevalence of anemia among HIV positive patients before and after initiation of ART

The prevalence of anemia in HIV patients was 41.9% (107/255) before ART initiation and 11.4% (29/255) after ART initiation. About 90 (84.1%) had mild anemia and 17 (15.9%) had moderate anemia before ART initiation.

From anemic patients after ART initiation, about 22 (75.9%) had mild anemia and 7 (24.1%) had moderate anemia. Severe anemia was not found in this study. The prevalence of anemia after ART initiation was significantly decreased by 30.5%.

### Risk factors of anemia in HIV infected patients before and after ART initiation

From anemic patients before ART initiation, about 43.9% (47/107) were males and 40.5% (60/148) were females. Similarly 42.5% (48/113) of patients were within the age of 30 to 40 years and 49.4% of patients with CD4 cell count < 200 cells/μl developed anemia. There were significant associations between anemia with CD4 cell count and WHO clinical stage before ART initiation. HIV patients with CD4 cell count < 200 cells/μl before ART initiation had higher prevalence of anemia (49.4%, *p* <  0.001). Similarly about 48.4% (*p* <  0.05) patients with WHO clinical stage III/IV had anemia before ART initiation. However, there was no significant association of anemia with sex and age (Table 3).

**Table 3:** Anemia and its associated factors before ART initiation in HIV positive patients attending Black Lion Specialized Hospital, Addis Ababa, Ethiopia, 2017

Variables	Anemic	Non anemic	X^2^	*P* value	AOR (95% CI)
Age(in years)					
<30	13 (39.4%)	20 (60.6%)	0.10	0.95	1.13 (0.49–2.63)
30–40	48 (42.5%)	65 (57.5%)			1.02 (0.58–1.78)
>40	46 (42.2%)	63 (57.8%)			1.00
Sex					
Male	47 (43.9%)	60 (56.1%)	0.21	0.65	1.09 (0.64–1.86)
Female	60 (40.5%)	88 (59.5%)			1.00
WHO clinical stages					
Stage I/II	32 (32%)	68 (68%)	6.70	**0.01**	1.00
Stage III/IV	75 (48.4%)	80 (51.6%)			1.83 (1.06–3.15)
CD4 count (cells/mm^3^)					
<200	89 (49.4%)	91 (50.6%)	14.07	< **0.001**	2.91 (1.57–5.39)
≥200	18 (24%)	57 (76%)			1.00

Numerical data in bold indicates the level of significance (*p* < 0.05), *AOR* Adjusted odds ratio, *CI* Confidence interval, 1.00 = reference group

Multiple logistic regression analysis was performed to obtain the adjusted effect of different risk factors on the odds of being anemic before initiation of ART. Table 3 summarizes the result of the final regression model. The variables; age, sex, WHO clinical stages and CD4 counts were included in the analysis. After adjusting for these factors in a multiple logistic regression analysis; clinical stage III/IV and CD4 count < 200 cells/mm^3^ were significantly associated with increased odds of being anemic.

From anemic patients after ART initiation, about 9.3% (10/107) were males and 12.8% (19/148) were females. Similarly 21.2% of patients with the age of < 30 years and 16.2% of patients with CD4 cell count < 200 cells/μl developed anemia. Although, HIV patients with CD4 cell count < 200 cells/μl had higher prevalence of anemia (16.2%) after ART initiation, there was no significant association between anemia and CD4 cell counts (*p* = 0.27). Similarly sex, age and ART regimen types had no significant association with anemia after ART initiation (Table 4). In multivariable logistic regression analysis, increased risk of anemia after 6 months of ART was observed among participants with age less than 30 years and CD4 count < 200 cells/mm^3^ whereas the odds of being anemic was lower among individuals with TDF based ART regimen, BMI 18.5–24.9 kg/m^2^ and male participants.

### Types of anemia among HIV positive patients before and after ART initiation

**Fig. 1:**
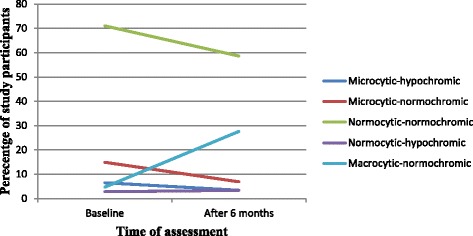
Types of anemia among HIV positive patients attending Black Lion Specialized Hospital, Addis Ababa, Ethiopia, 2017

From the total anemic HIV infected patients at the baseline, 71% had normocytic-normochromic anemia followed by microcytic-normochromic anemia 14.9%. After 6 months of ART initiation, normocytic-normochromic anemia was present in 58.6% of the cases followed by macrocytic-normochromic anemia in 27.6% of the cases (Fig. 1).

**Table 4:** Anemia and its associated factors after ART initiation in HIV positive patients attending Black Lion Specialized Hospital, Addis Ababa, Ethiopia, 2017

Variables	Anemia	Non anemic	X^2^	*P* value	AOR (95% CI)
Age(in years)					
<30	7 (21.2%)	26 (78.8%)	6.23	0.05	4.01 (1.24–13.02)
30–40	15 (13.3%)	98 (86.7%)			2.25 (0.84–5.98)
>40	7 (6.4%)	102 (93.6%)			1.00
Sex					
Male	10 (9.3%)	97 (90.7%)	1.19	0.28	0.79 (0.34–1.85)
Female	19 (12.8%)	129 (87.2%)			1.00
ART regimen					
TDF based	21 (11.4%)	164 (88.6%)	0.000	0.99	0.99 (0.40–2.46)
AZT based	8 (11.4%)	62 (88.6%)			1.00
Cotrimoxazole					
NO	9 (8.9%)	92 (91.1)	1.01	0.32	0.74 (0.29–1.86)
Yes	20 (13%)	134 (87)			1.00
CD4 count (cells/mm^3^)					
<200	10 (16.2%)	52 (83.9%)	2.62	0.27	2.04 (0.70–5.96)
200–349	10 (12.2%)	72 (87.8%)			1.44 (0.52–3.99)
≥350	9 (8.1%)	102 (91.9%)			1.00
BMI(Kg/m^2^)					
<18.5	4 (12.9%)	27 (87.1%)	0.08	0.96	0.81 (0.21–3.17
18.5–24.9	18 (11.2%)	143 (88.8%)			0.76 (0.29–2.01)
≥25	7 (11.1%)	56 (88.9%)			1.00

*AOR* Adjusted odds ratio, *BMI* Body mass index, *CI* Confidence interval, 1.00 = reference group

## Discussion

Anemia is the most common hematological abnormality and it has been associated with an increased HIV disease progression among HIV infected patients [13]. As recovery from anemia led to improvements in patient survival, screening for anemia among HIV infected patients should be performed to decrease the risk of death and to enhance the individual’s functional status [4]. All subjects in this study were under first line of antiretroviral therapy.

The present study revealed that the prevalence of anemia at the baseline was 41.9% with 84.1% mild and 15.9% moderate anemia. After 6 months of ART, the prevalence of anemia was reduced to 11.4%. This finding is in agreement with a study done in Addis Ababa, Ethiopia reported that the prevalence of anemia at the baseline was 42.9% with 79, 15.6 and 5.3% mild, moderate and severe anemia respectively. The study also added that at 12 months of ART initiation the prevalence of anemia was reduced to 14.3% [11]. In a separate study at Arba Minch, Ethiopia the prevalence of anemia at baseline was 52.3% with 28.1, 22.9 and 1.3% mild, moderate and severe anemia respectively [4]. Other studies also found different prevalence rates of anemia. A study from Hawassa, Ethiopia reported the prevalence of anemia as 23.4% before ART and 12.0% after ART [14]. A study from Jimma, Ethiopia reported the prevalence as 29.9% before ART and 16.2% after ART [13]. The baseline prevalence of anemia reported from Hawassa and Jimma is lower than the present study. On the other hand, a study from North eastern Nigeria stated a prevalence rate of anemia in ART-naive patients was 57.7% and it was reduced to 24.3% in ART-experienced patients [15]. The reasons for the observed differences might be due to the difference in the study population, sample size, study design and variability in the definition of anemia.

The decrease in the prevalence of anemia after ART initiation is attributed to the positive effect of ART on the differentiation and survival of erythrocytes. HIV infection of marrow stromal cells, decrease in serum erythropoietin levels, auto-antibodies to erythropoietin, or marrow suppression by opportunistic infections, may contribute to the anemia commonly observed in HIV-infected patients. ART may ameliorate many of these effects in an indirect manner simply by decreasing the HIV viral burden [15].

In this study, the prevalence of anemia observed across sex groups was higher in men than in women before ART initiation (43.9% vs 40.5%), and lower after ART initiation (9.3% vs. 12.8%). In agreement with this finding, a report from Benin city, Nigeria showed that among ART naive HIV patients, men had higher prevalence of anemia than their women counterparts (76.42% vs 63.43%), and lower in those patients on ART (44.19% vs 55.73%) [16]. The findings in this study differ from the findings of a study in Hawassa, Ethiopia [14] and Addis Ababa, Ethiopia [17]. Female gender has been reported as a risk factor for anemia among HIV patients [8]. In the present study, the same finding was obtained among HIV patients who were receiving ART. Although statistically insignificant, the prevalence of anemia post ART initiation was dropped from 43.9 to 9.3% in men and from 40.5 to 12.8% in women.

We found that, the prevalence of anemia was increased with decreasing CD4 count both before and after ART initiation with a high prevalence among patients with CD4 count < 200 cells/mm^3^. In agreement with this finding, various studies reported that anemia was more prevalent among HIV patients with CD4 count < 200 cells/mm^3^ [3, 4, 11, 18]. In general, in the advanced stage of the disease, the blood cell counts were lower than the early stage of the diseases. This might be due to the increasing trend in the frequency of bone marrow abnormalities with progressive immunologic deterioration and advanced disease due to HIV [19, 20].

The types of anemia were also assessed in this study. Among the total number of anemic cases, normocytic normochromic anemia was present in 71 and 58.6% of the cases before and after ART initiation respectively. A study conducted in Gondar, Ethiopia reported that the prevalence of normocytic normochromic anemia in ART naive patients was 48.9%, which reduced to 29.4% in those patients on ART [21]. This difference might be due to a difference in the definition of the types of anemia.

In the present study the relatively higher risk of developing microcytic hypochromic anemia was found in HIV positive patients before ART as compared to those on ART. Another cross sectional study in Ghana showed that, the likelihood of developing microcytic hypochromic anemia in ART-naive patients was five times more compared to those on ART [22]. This may reflect the overall nutritional deficiencies (malnutrition and malabsorption) associated with HIV patients.

This study also found that macrocytic normochromic anemia was present in 4.7% of anemic subjects at the baseline which was increased to 27.6% after ART initiation. This showed that the average MCV for patients on ART were significantly higher compared to their ART naïve patients and the degree of macrocytosis is more to the group receiving zidovudine. In agreement with the current findings, studies conducted in Ghana [22] and Ethiopia [21] reported that macrocytic normochromic anemia was more common after ART than before ART initiation. This is probably due to the effect of ART particularly AZT which is responsible for the development of macrocytosis.

This study assesses the prevalence of anemia before and after initiation of ART. However, this study had limitations such as lack of age and sex matched healthy control group. Also the study does not address viral load and albumin levels because of lack of resources. In addition, this study did not include baseline data on BMI. Nevertheless, this study provides valuable information about the burden of anemia among HIV positive patients before and after ART.

## Conclusions

There was a remarkable reduction in the prevalence of anemia after ART initiation. WHO clinical stages and CD4 + T cell counts were associated with the prevalence of anemia before ART initiation. Normocytic normochromic anemia was the commonest type of anemia before and after ART initiation. Based on the present findings, a significant proportion of HIV patients remained anemic after 6 months of ART initiation suggesting the need for routine screening and proper treatment of anemia to mitigate its adverse effects.

## Abbreviations

3TC: Lamivudin
AIDS: Acquired immunodeficiency syndrome
ART: Antiretroviral treatment
AZT/ZDV: Azidothymidine/Zidovudine
BMI: Body mass index
CD4: Cluster of differentiation 4
EVF: Efavirenz
Hgb: Hemoglobin
HIV: Human immunodeficiency virus
MCH: Mean cell hemoglobin
MCHC: Mean cell hemoglobin concentration
MCV: Mean cell volume
NVP: Nevirapine
RBC: Red blood cell
RDW: Red cell distribution width
TDF: Tenofovir
